# The Prognostic Significance of Quantitative Myocardial Perfusion: An Artificial Intelligence–Based Approach Using Perfusion Mapping

**DOI:** 10.1161/CIRCULATIONAHA.119.044666

**Published:** 2020-02-14

**Authors:** Kristopher D. Knott, Andreas Seraphim, Joao B. Augusto, Hui Xue, Liza Chacko, Nay Aung, Steffen E. Petersen, Jackie A. Cooper, Charlotte Manisty, Anish N. Bhuva, Tushar Kotecha, Christos V. Bourantas, Rhodri H. Davies, Louise A.E. Brown, Sven Plein, Marianna Fontana, Peter Kellman, James C. Moon

**Affiliations:** 1 Institute of Cardiovascular Science, University College London, United Kingdom (K.D.K., A.S., J.B.A., L.C., C.M., A.N.B., T.K., C.V.B., R.H.D., M.F., J.C.M.).; 2 Barts Heart Centre, St Bartholomew’s Hospital, London, United Kingdom (K.D.K., A.S., J.B.A., N.A., S.E.P., C.M., A.N.B., C.V.B., R.H.D., J.C.M.).; 3 National Heart, Lung, and Blood Institute, National Institutes of Health, Department of Health and Human Services, Bethesda, MD (H.X., P.K.).; 4 Royal Free Hospital, London, United Kingdom (L.C., T.K., M.F.).; 5 William Harvey Research Institute, Queen Mary University of London, United Kingdom (N.A., S.E.P., J.A.C.).; 6 Department of Biomedical Imaging Science, Leeds Institute of Cardiovascular and Metabolic Medicine, University of Leeds, United Kingdom (L.A.E.B., S.P.).

**Keywords:** cardiovascular magnetic resonance, cardiovascular outcomes, inline perfusion quantification, myocardial perfusion

## Abstract

**Background:**

Myocardial perfusion reflects the macro- and microvascular coronary circulation. Recent quantitation developments using cardiovascular magnetic resonance perfusion permit automated measurement clinically. We explored the prognostic significance of stress myocardial blood flow (MBF) and myocardial perfusion reserve (MPR, the ratio of stress to rest MBF).

**Methods:**

A 2-center study of patients with both suspected and known coronary artery disease referred clinically for perfusion assessment. Image analysis was performed automatically using a novel artificial intelligence approach deriving global and regional stress and rest MBF and MPR. Cox proportional hazard models adjusting for comorbidities and cardiovascular magnetic resonance parameters sought associations of stress MBF and MPR with death and major adverse cardiovascular events (MACE), including myocardial infarction, stroke, heart failure hospitalization, late (>90 day) revascularization, and death.

**Results:**

A total of 1049 patients were included with a median follow-up of 605 (interquartile range, 464–814) days. There were 42 (4.0%) deaths and 188 MACE in 174 (16.6%) patients. Stress MBF and MPR were independently associated with both death and MACE. For each 1 mL·g^-1^·min^-1^ decrease in stress MBF, the adjusted hazard ratios for death and MACE were 1.93 (95% CI, 1.08–3.48, *P*=0.028) and 2.14 (95% CI, 1.58–2.90, *P*<0.0001), respectively, even after adjusting for age and comorbidity. For each 1 U decrease in MPR, the adjusted hazard ratios for death and MACE were 2.45 (95% CI, 1.42–4.24, *P*=0.001) and 1.74 (95% CI, 1.36–2.22, *P*<0.0001), respectively. In patients without regional perfusion defects on clinical read and no known macrovascular coronary artery disease (n=783), MPR remained independently associated with death and MACE, with stress MBF remaining associated with MACE only.

**Conclusions:**

In patients with known or suspected coronary artery disease, reduced MBF and MPR measured automatically inline using artificial intelligence quantification of cardiovascular magnetic resonance perfusion mapping provides a strong, independent predictor of adverse cardiovascular outcomes.

## Abstract 2

Supplemental Digital Content is available in the text.

**Clinical Perspective**

**What Is New?**

- Perfusion mapping uses artificial intelligence to provide instantaneous quantification of myocardial perfusion by cardiovascular magnetic resonance.
- Quantitative myocardial blood flow provides incremental prognostic information in patients with suspected coronary artery disease above traditional cardiovascular risk factors.
- Even in patients without regional perfusion defects, absolute perfusion is prognostic.

**What Are the Clinical Implications?**

- Absolute perfusion quantification is a likely new biomarker in patient care.
- As there is no user input and no ionizing radiation, early disease and microvascular disease can be studied at scale.
- Impaired global perfusion may be a targetable cardiovascular risk factor.

**Editorial, see p 1292**

Cardiovascular disease is the leading global cause of mortality and morbidity,^1^ with chronic coronary syndromes a leading contributor. Chronic coronary syndromes include macrovascular epicardial coronary artery disease (CAD) and microvascular dysfunction,^2^ both of which result in reduced myocardial blood flow and adverse outcomes^3^ but are amenable to medical and interventional therapies.^4^ Invasive assessment strategies (fractional flow reserve [FFR] and the index of microcirculatory resistance)^5,6^ to measure blood flow are now recommended by international guidelines, but these are associated with risk.^7,8^ Noninvasive, functional perfusion testing has not superseded this strategy because it has not yet demonstrated sufficient prognostic importance and is frequently assessed qualitatively.

Functional perfusion tests include positron emission tomography (PET), single photon emission tomography, and cardiovascular magnetic resonance (CMR). All are accurate for the detection of epicardial CAD,^9^ but by measuring tissue blood flow, in addition they capture microvascular disease, which is an advantage for understanding the whole myocardial circulation. Using PET, absolute quantification of myocardial blood flow (MBF) and the ratio of stress to rest MBF, known as the myocardial perfusion reserve (MPR) or coronary flow reserve (CFR), can be performed. Quantitative PET perfusion encodes prognostic information in suspected chronic coronary syndromes^10–13^ and cardiomyopathy^14^ and is potentially less operator dependent and less likely to miss balanced ischemia than qualitative techniques.

An alternative to PET that does not use ionizing radiation is CMR. Stress perfusion CMR has been validated against intracoronary blood flow for detecting CAD,^15,16^ with death and major adverse cardiovascular events (MACE) at 1 year being similar between patients managed initially with stress perfusion CMR- or FFR-guided strategies.^17^ Unlike PET perfusion, CMR has been primarily qualitative to date because of the complexity and time needed for quantitation. This is now changing with the development of new quantitative techniques. “Perfusion mapping” is an approach where, in addition to conventional images, perfusion maps are generated automatically on the scanner with each image pixel encoding MBF (mL·g^-1^·min^-1^).^18^ The technique has been validated in healthy volunteers against PET, coronary angiography, and invasive physiology and provides insight into microvascular function in cardiomyopathy.^19–23^ The latest software iterations using artificial intelligence approaches deliver automatic segmental and global quantitation, permitting efficient large-scale analysis. These artificial intelligence approaches have been applied to volume analysis in CMR and have the potential to provide precise, rapid image biomarkers of cardiac structure and function^24^ but have not been applied to perfusion imaging before.

We aimed to investigate whether, in a multicenter setting including all-comers, quantitative myocardial perfusion (global mean stress MBF and MPR) by CMR perfusion mapping would be independently associated with adverse outcomes.

## Methods

All included data for this study are available from the corresponding author upon reasonable request.

### Patients

The study was approved by the National Health Service Research Ethics Committee and Health Research Authority and conducted in accordance with the Declaration of Helsinki (Barts Bioresource—REC ID 14/EE/0007, Royal Free Hospital—REC ID 07/H0715/101). We included consecutive patients age 18 years and older referred to 2 centers (Barts Heart Centre and the Royal Free Hospital, London, United Kingdom), between March 2016 and August 2018 for stress perfusion CMR and who had provided written, informed consent and had >1 year follow-up available. We excluded patients who were diagnosed with inherited or infiltrative cardiomyopathies known to affect myocardial perfusion (such as hypertrophic cardiomyopathy and cardiac amyloid) from the analysis.

Patient comorbidities and outcomes were documented from the electronic patient record and the National Health Service Spine portal. Comorbidities recorded were previous revascularization (percutaneous coronary intervention or coronary artery bypass graft), CAD, hypertension, dyslipidemia, diabetes mellitus, atrial fibrillation, stroke or transient ischemic attack, smoking, and cancer. The study outcomes were all-cause mortality and a composite of major adverse cardiovascular events (defined as myocardial infarction, stroke, heart failure admission, revascularization, or death). Revascularization events <90 days after CMR were excluded to prevent the inclusion of events occurring as a result of the perfusion CMR. MACE was adjudicated by a committee of 3 cardiologists blinded to the perfusion data.

### Cardiovascular Magnetic Resonance Scan

All scans were performed at 1.5 (Aera) or 3 Tesla (Prisma, Siemens Healthcare, Erlangen, Germany) according to a standard protocol including cine imaging, adenosine stress and rest perfusion, and late gadolinium enhancement (LGE). Patients were asked to abstain from caffeine for 24 hours before the scan. All patients underwent adenosine stress according to a standard clinical protocol.^25^ Adenosine was infused at 140 mcg/kg/min for 4 minutes. If there were no symptoms and no ≥10 beat per minute heart rate increase, the infusion rate was increased to 175 mcg/kg/min.^26,27^ At maximal hyperemia, a gadolinium-based contrast agent (gadoterate meglumine, Dotarem, Guerbet, Paris, France) was injected at 4 mL/s at a dose of 0.05 mmol/kg. Perfusion maps were generated automatically inline at the time of the scan according to Kellman et al.^18^ The acquisition was repeated at rest 5 to 10 minutes later (after the short axis stack).

### Image Analysis

All CMR studies were analyzed by a cardiologist accredited by the European Association of Cardiovascular Imaging or Society of Cardiovascular Magnetic Resonance (level 3). Image analysis was performed using commercially available software (CVI42, Circle Cardiovascular Imaging, Calgary, Alberta, Canada). Left ventricular systolic and diastolic volume, ejection fraction, and the presence and distribution (infarct or noninfarct) of LGE were recorded.

**Figure 1.**
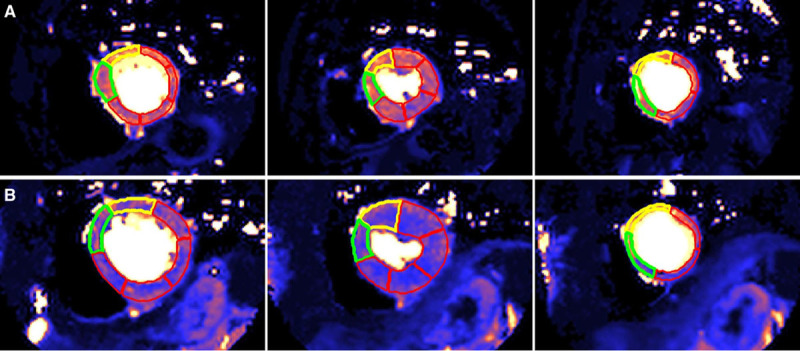
**Automatic segmentation of the stress perfusion maps performed by machine learning with no user input.** Base, mid, and apical left ventricle short axis slices (left to right) for a 76-year-old man with dyslipidemia and no death or major adverse cardiovascular events (**A**) and a 64-year-old woman with hypertension and atrial fibrillation who died within 24 months of the scan (**B**). Mean stress myocardial blood flow was 2.25 mL·g^-1^·min^-1^ in (**A**) and 1.52 mL·g^-1^·min^-1^ in (**B**).

Perfusion maps (3 short axis slices per patient) were generated automatically and inline at the time of the scan as described by Kellman et al.^18^ The perfusion sequence is a dual sequence technique^28^ whereby there is a low-resolution arterial input function acquisition and a high-resolution myocardial perfusion acquisition simultaneously. Dual sequence perfusion quantification has been validated against microspheres.^29^ Perfusion is quantified for each pixel of myocardium,^18^ and perfusion maps are generated within 90 s of the scan. Each pixel encodes the myocardial blood flow (mL·g^-1^·min^-1^). The artificial intelligence tool performs automatic segmentation of the left ventricle cavity and myocardium. It uses a convolution neural net approach to delineate the left ventricle cavity and myocardium, excluding myocardial fat and papillary muscles.^30^ The global MBF is then calculated automatically as an average of all pixels and global MPR as the ratio of stress to rest MBF. As they were contoured without user input, the perfusion data were blinded to other CMR and demographic parameters (Figure 1). Contoured perfusion maps were subsequently visually inspected by an observer (blinded to other parameters and outcome data) for quality control and discarded if there were errors. After automatic artificial intelligence contouring, no human modification of contours was performed on any of the perfusion maps. The global mean stress MBF, rest MBF, and MPR were recorded.

### Statistical Analysis

Statistical analysis was performed in SPSS (IBM SPSS Statistics, version 25.0). Continuous variables are presented as mean±SD or median±interquartile range for normal and nonnormally distributed data, respectively. Categorical variables are presented as absolute values and percentages. Means were compared using the Student *t* test or Mann-Whitney *U* test (depending on normality) for continuous variables and χ^2^ test (2-sided Fisher exact test) for categorical variables. A *P* value of <0.05 was considered statistically significant.

A Cox proportional hazard regression analysis was performed to determine whether perfusion data (stress MBF and MPR) were associated with death and MACE adjusting for age, sex, comorbidities (previous revascularization, CAD, hypertension, dyslipidemia, diabetes mellitus, atrial fibrillation, stroke or transient ischemic attack, smoking, and cancer) and CMR parameters (end diastolic volume, left ventricular ejection fraction, LGE). A sensitivity analysis using a penalized model was performed to obtain the Firth bias-adjusted estimates to ensure there was no bias in the estimated coefficients caused by low event rates.^31^ Kaplan-Meier survival estimates were then plotted for the upper and lower 50th percentiles of stress MBF and MPR. Harrel C-indices were used to compare the relative predictive ability of stress MBF and MPR. For this analysis, the data were censured at the date of death, MACE, or last follow-up.

The proportionality assumption was tested using the Schoenfeld residuals. The assumption was tested for each individual variable using a Bonferroni-corrected significance level of *P*<0.0008. Functional form was assessed by plotting deviance residuals against each predictor variable and assessing the locally estimated scatterplot smoothing curve. Models were run with and without imputation of missing data. Both analyses gave similar results, and only complete case results are shown. Multiple imputation by chained equations was used to impute 10 complete datasets, and results were pooled. Predictive mean matching with the 5 nearest neighbors was used for continuous variables and logistic regression for binary variables. All variables used in the analysis models were included in the imputation.

## Results

### Patient Demographics, Comorbidities, and CMR Parameters

**Figure 2.**
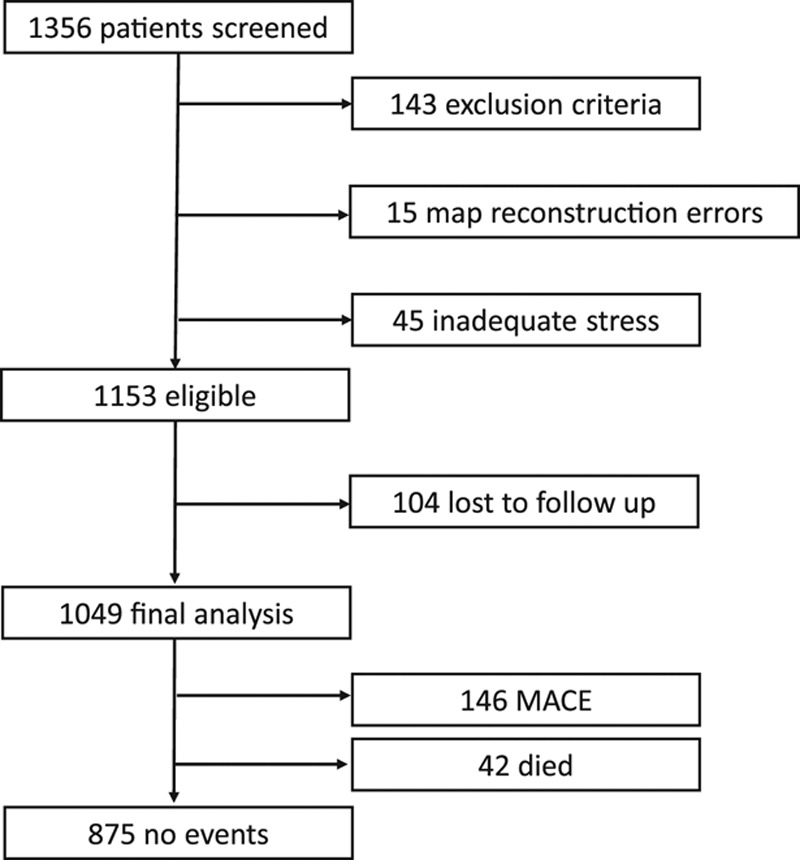
**Study flow chart.** A total of 1049 patients were included in the final analysis. A total of 143 patients met the exclusion criteria, there were reconstruction errors in perfusion maps in 15 cases, and there were 45 cases of inadequate stress (no splenic switch off). A total of 104 patients were lost to follow-up. There were 188 events in total (major adverse cardiovascular events [MACE]) in 174 patients, including 42 deaths.

A total of 1356 eligible patients were referred for stress perfusion CMR at Barts Heart Centre and the Royal Free Hospital between September 2016 and August 2018. Of these, 143 patients met the exclusion criteria, and in 45 (3%) patients, there was no apparent stress response through heart rate, symptoms, splenic switch off, or myocardial vasodilatation, so we excluded these. A total of 15 (1%) had perfusion map errors, preventing analysis. A total of 104 (8%) patients were lost to follow-up. In total, 1049 patients were included (889 from Barts Heart Centre, 160 from Royal Free Hospital, Figure 2). In 31 patients, rest perfusion was not performed, so 1018 patients had MPR data.

The mean age of patients was 60.9±13 years, 702 (67%) were men, 298 (28%) had diabetes mellitus, 630 (60%) had hypertension, 510 (49%) had dyslipidemia, 318 (30%) had previous revascularization, 360 (34%) had smoking history, 63 (6%) had previous stroke or transient ischemic attack, 141 (13%) had atrial fibrillation, and 108 (10%) had a current or previous history of cancer. The mean ejection fraction was 60±13%, and 309 (30%) patients had infarct pattern and 133 (13%) noninfarct pattern LGE. Patient characteristics and CMR findings are summarized in Table 1. Mean stress MBF was 2.06±0.71 mL·g^-1^·min^-1^, and mean MPR was 2.48±0.82.

**Table 1.**
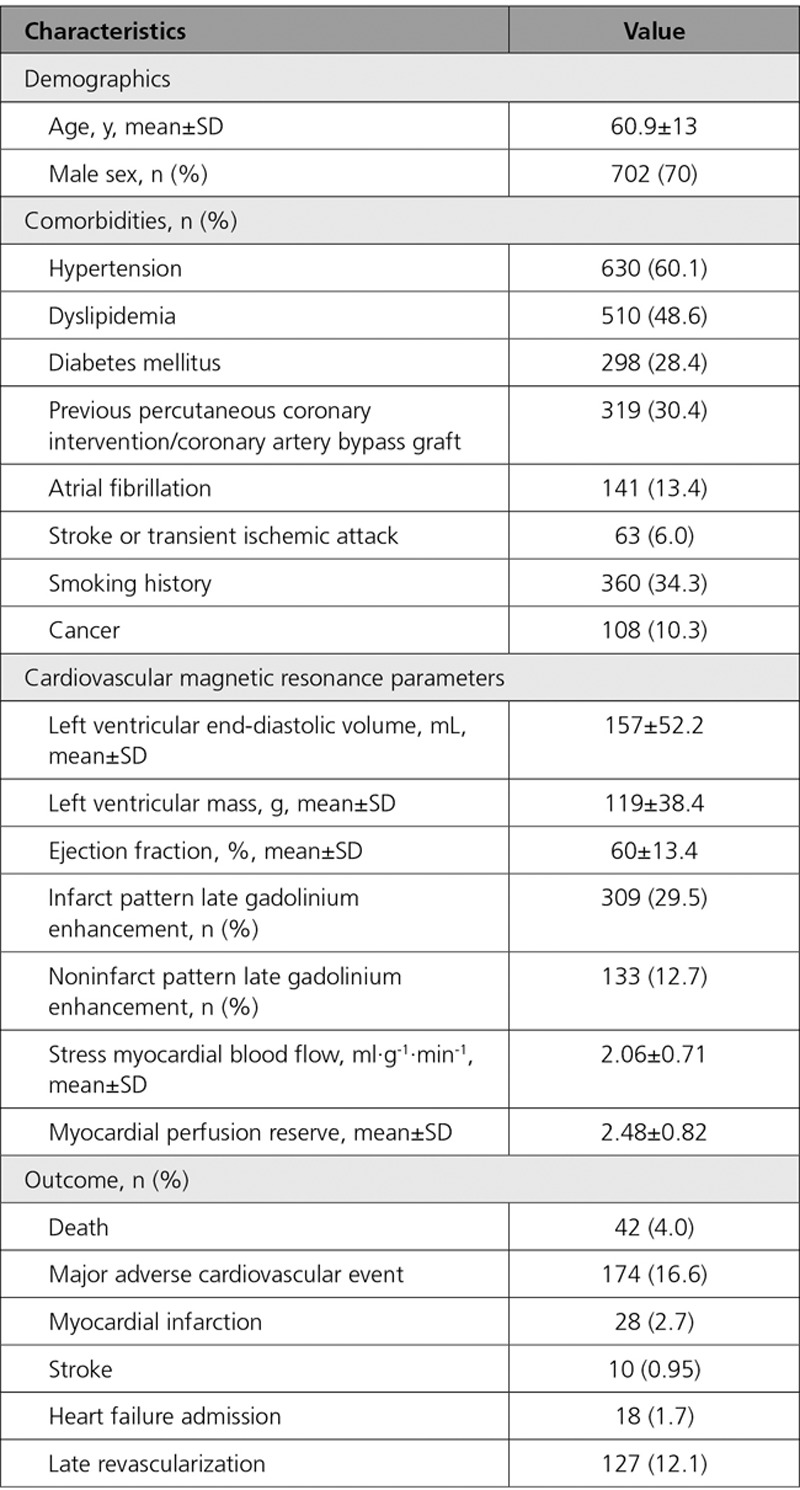
Baseline Demographics, Cardiovascular Magnetic Resonance Parameters, and Outcomes of the Study Population (N=1049)

### Predictors of MACE

There were 42 (4.0%) deaths during a median follow-up period of 605 (interquartile range, 464–814) days. In total, there were 188 MACEs in 174 (16.6%) patients. This included 28 (2.7%) myocardial infarctions, 10 (0.95%) strokes, 18 (1.7%) heart failure admissions, and 127 (12.1%) late revascularizations. MBF was lower in those who died (1.70±0.65 versus 2.08±0.71 mL·g^-1^·min^-1^, *P*=0.001), as was MPR (1.97±0.74 versus 2.50±0.81, *P*<0.001). Similar reductions occurred for total events (death or MACE) for both MBF and MPR (both *P* values <0.001).

Patients who had a MACE were more commonly men, were older, more often had previous revascularizations, and were more likely to have diabetes mellitus, hypertension, dyslipidemia, a previous stroke or transient ischemic attack, and a positive smoking history. In addition, they had a lower left ventricular ejection fraction and more often an infarct pattern LGE (Table 2). For a breakdown of perfusion data and MACE for each site and field strength, see Tables I and II in the Data Supplement.

**Table 2.**
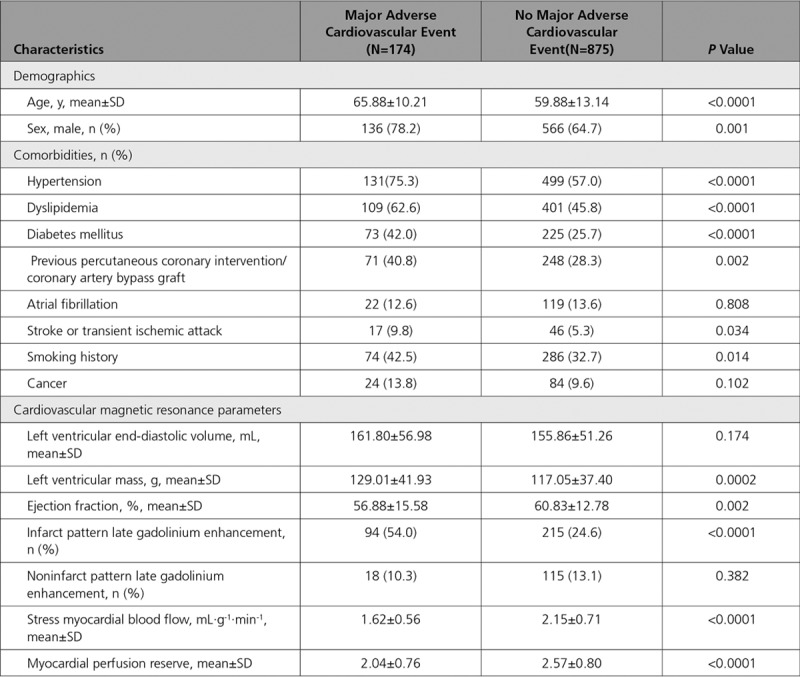
A Comparison Between Patients Who Had Died or Had a Major Adverse Cardiovascular Event and Those Who Did Not

**Figure 3.**
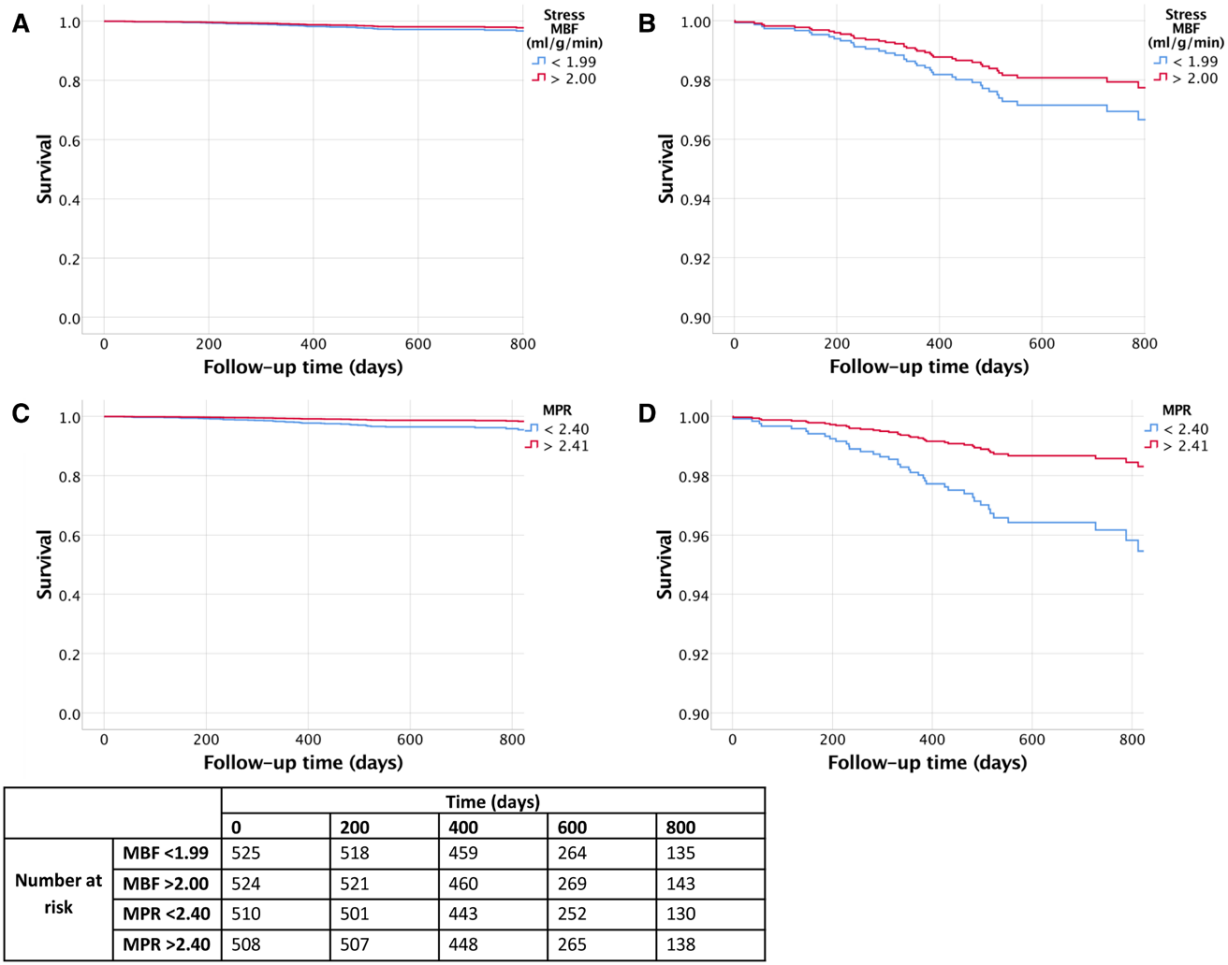
**Kaplan-Meier survival estimate curves for stress myocardial blood flow (MBF) and myocardial perfusion reserve (MPR).** Stress MBF (**A** and **B**) and MPR (**C** and **D**). The red lines demonstrate the survival curves for the highest 50th percentile, and the blue lines demonstrate the lowest 50th percentile of patients. **B** and **D**, Magnified to highlight the separation of the curves. Rates of death are higher with impaired perfusion. Compared with patients in the highest 50th percentile, the patients in the lowest 50th percentile of MBF and MPR had higher rates of death (*P*=0.032 and *P*=0.01, respectively).

**Figure 4.**
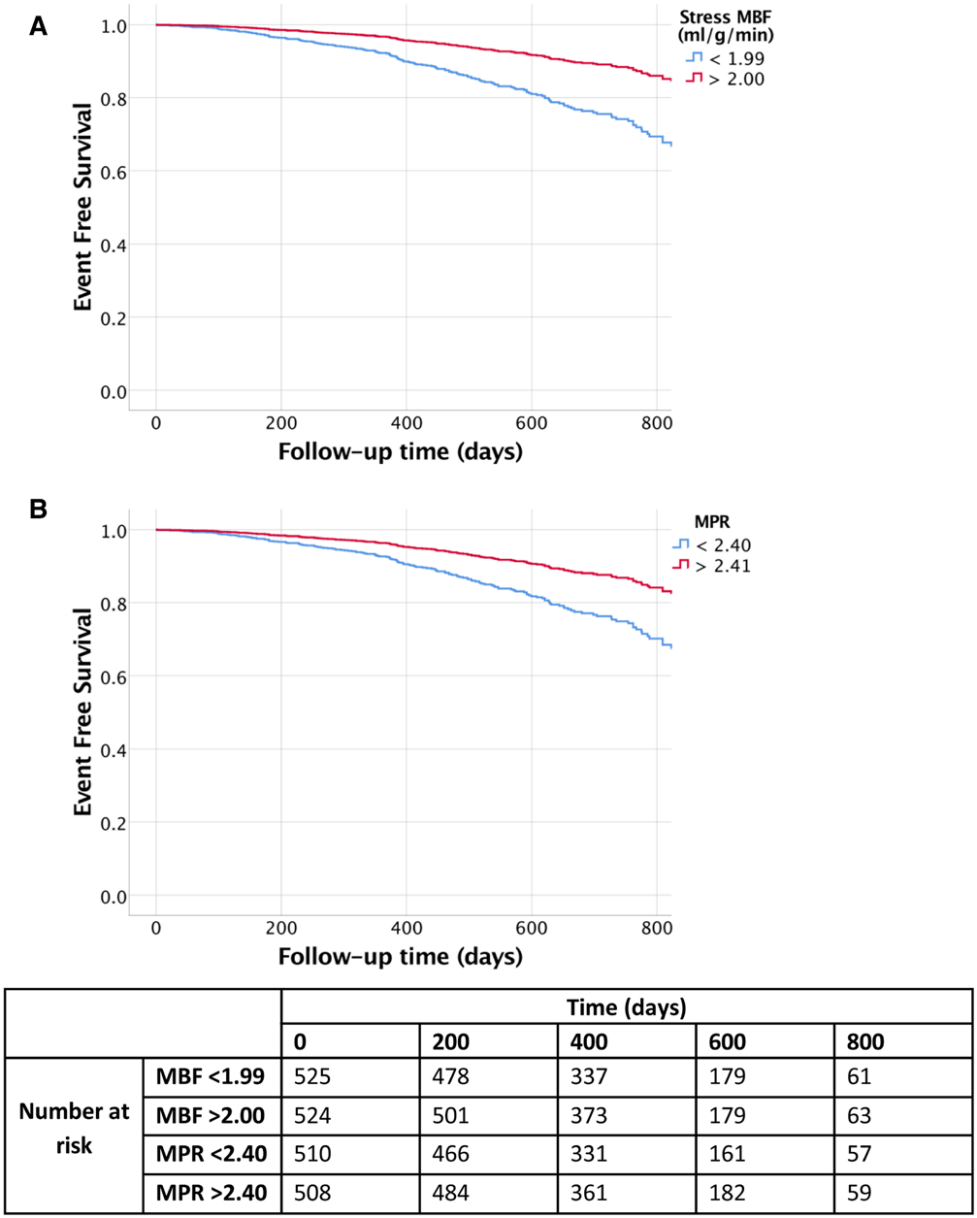
**Kaplan-Meier survival estimate curves for stress myocardial blood flow (MBF) and myocardial perfusion reserve (MPR).** The Kaplan Meier survival estimate curves demonstrate major adverse cardiovascular events over time for stress MBF (**A**) and MPR (**B**). The red lines demonstrate the survival curves for the highest 50th percentile, and the blue lines demonstrate the lowest 50th percentile of patients. Compared with patients in the highest 50th percentile, the patients in the lowest 50th percentile of MBF and MPR had higher rates of death (*P*<0.001 for both).

Cox hazard regression analysis demonstrated that stress MBF and MPR were associated with events after adjusting for potential confounders. The adjusted hazard ratio (HR) for 1 mL·g^-1^·min^-1^ decrease in stress MBF was 1.93 (95% CI, 1.08–3.48) for death (*P*=0.028) and 2.14 for MACE (95% CI, 1.58–2.90, *P*<0.0001). The adjusted HR for a 1 U decrease in MPR was 2.45 (95% CI, 1.42–4.24) for death (*P*=0.001) and 1.74 (95% CI, 1.36–2.22) for MACE (*P*<0.0001, Table 3). A standardized hazard model found the effect of MPR to be larger than stress MBF for death (standardized HR for a 1 SD reduction in MPR or MBF, 2.08 versus 1.56, respectively), but not for death or MACE (standardized HR, 1.59 versus 1.79). The predictive ability for MPR (C-index, 0.69 [95% CI, 0.61–0.77]) was better than for MBF (C-index, 0.63 [95% CI, 0.54–0.73]) when predicting death, but both variables had similar predictive ability for the death or MACE (0.68 [95% CI, 0.64–0.73] MBF versus 0.68 [95% CI, 0.64–0.72] MPR). A sensitivity analysis did not indicate any bias caused by low event rates. Kaplan-Meier survival estimate curves for MBF and MPR are presented in Figure 3 (death) and Figure 4 (MACE). Death or MI was associated with stress MBF and MPR, age, LGE, and history of cancer. The adjusted HR for a 1 mL·g^-1^·min^-1^ decrease in MBF was 2.32 (95% CI, 1.43–3.77) and for a 1 U decrease in MPR was 2.63 (95% CI, 1.70–4.10).

**Table 3.**
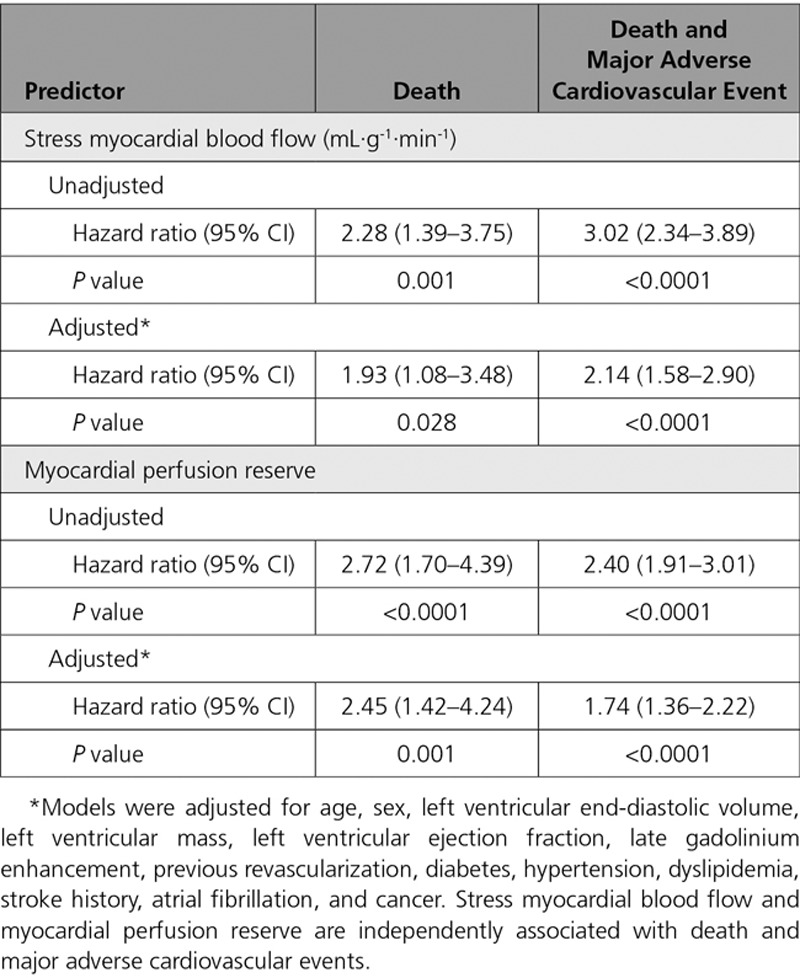
Cox Proportional Hazard Models for a 1 mL·g^-1^·min^-1^ Decrease in Stress Myocardial Blood Flow and 1 U Decrease in Myocardial Perfusion Reserve

In total, 266 patients (25.4%) had a regional perfusion defect on clinical read in a least 1 myocardial segment. Deaths were no different between patients with regional perfusion defects and “normal” (uniform) perfusion (14 [5.3%] versus 28 [3.6%], *P*=0.276), but MACE was higher (103 [39%] versus 71 [9.1%], *P*<0.0001). Mean global stress MBF and MPR were lower in the perfusion defect group (1.74±0.62 mL·g^-1^·min^-1^ versus 2.17±0.71 mL·g^-1^·min^-1^, *P*<0.0001 and 2.14±0.75 and 2.59±0.81, *P*<0.0001, respectively).

A multivariate Cox regression analysis was also performed on patients with no regional perfusion defects. Death was associated with, age, ejection fraction, history of cancer, history of hypertension, and MPR but not stress MBF. MACE was associated with age, a history of cancer, and both stress MBF and MPR. The adjusted HR for a 1 U decrease in MPR was similar to the whole cohort: 2.22 (95% CI, 1.16–4.23) for death (*P*=0.015) and 1.65 (95% CI, 1.14–2.38) for MACE (*P*=0.008) with stress MBF HR of 2.28 (95% CI, 1.43–3.66) for MACE (*P*=0.001).

A further Cox regression analysis was performed excluding patients with previous CAD, myocardial infarction, or LGE. Death was associated with age, a history of cancer, dyslipidemia, and MPR. MACE was associated with age, a history of cancer, and both stress MBF and MPR. The adjusted HR for a 1 U decrease in MPR was 2.49 (95% CI, 1.01–6.13) for death (*P*=0.049) and 2.38 (95% CI, 1.30–3.77) for MACE (*P*=0.003) with stress MBF HR of 2.15 (95% CI, 1.20–3.83) for MACE (*P*=0.010).

## Discussion

This multicenter study, the largest quantitative perfusion CMR study to date, shows that myocardial stress MBF and MPR by CMR perfusion mapping are associated with adverse outcomes over and above other cardiovascular risk factors. This quantitation is possible in routine practice automatically at scale using an artificial intelligence–based approach, and these values are prognostic—a 1 SD increase in stress MBF (0.71 mL·g^-1^·min^-1^) or MPR (0.82) is associated with a reduced risk of death by 36% and 52% and MACE by 54% and 37%, even after adjusting for other risk factors. The ease of measurement and quantitation makes this attractive both clinically and for research as an end point in studies exploring therapy to improve perfusion.

This study confirms the prognostic relevance of myocardial perfusion, which has previously been shown in PET studies.^10–13^ For example, Herzog and colleagues followed up 256 patients for a mean of 5.4 years.^10^ They found that even in patients with no perfusion defects, an abnormal MPR (<2) was associated with worse outcomes. Perfusion CMR has several additional benefits. First, the spatial resolution is superior to other functional imaging modalities, reducing partial volume effects and improving the detection of perfusion abnormalities. CMR also does not use ionizing radiation, which is advantageous particularly for repeat studies. Furthermore, after perfusion, LGE images are acquired, which allows direct comparison of ischemia and infarction and allows the operator to discriminate between reversible and “matched” perfusion defects corresponding to infarct.

The quantitative approach here, perfusion mapping, has already been validated against both rubidium PET^20^ and invasive coronary physiology.^22^ Engblom et al recruited patients with stable CAD for PET and CMR perfusion on the same day. They showed that there was a good correlation with global (*r*=0.92) and regional flow (*r*=0.83).^20^ Kotecha et al studied invasive coronary physiology with FFR and index of microcirculatory resistance. They found that myocardium supplied by coronary arteries with FFR-positive lesions had significantly lower MBF and MPR than remote myocardium and that myocardium supplied by FFR-negative, index of microcirculatory resistance–positive lesions had intermediate perfusion.^22^ Brown et al found that the repeatability of perfusion mapping is similar to the published PET literature in a cohort of healthy volunteers.^19^ Our study adds weight to those validations by demonstrating prognostication in addition.

There has been 1 previous fully quantitative perfusion outcome CMR study, a single-center dual bolus study of 395 patients followed for a median 460 days. This found that decreased myocardial perfusion reserve, determined by a set threshold (1.5) of MPR for ischemia per segment with total number of segments summed, contained prognostic information for a composite MACE end point.^32^

The present study exploits recent CMR technical developments via a clinically feasible dual sequence approach with a pixelwise rather than segmental approach, and full automation of analysis making a multicenter approach with 3.5 times greater follow-up (1735 patient-years) feasible. It has also permitted the prognostic significance of MPR and MBF to be explored independently of other factors with multivariate modeling, placing CMR on the same footing as PET for ease of full blood flow quantification. For the first time, we have shown that automatically derived MBF and MPR have prognostic relevance beyond the detection of regional ischemia. This provides the opportunity for quantitative perfusion analysis to be applied in the routine clinical setting to potentially risk stratify beyond the detection of regional ischemia alone. The predictive power is moderate but incremental over conventional factors.

With a relatively small number of events, our finding that MPR may be superior to stress MBF in predicting death but not death or MACE should not be overstated. However, PET studies have also suggested MPR is a stronger predictor of cardiovascular mortality than maximal MBF. For example, Gupta et al found that CFR was a stronger predictor of cardiovascular death than MBF in a study of 4029 patients with a median 5.6-year follow-up.^33^ Patients with impaired CFR and MBF had the worst prognosis, and the best outcome was when CFR and MBF were both normal. When the MBF was abnormal but the CFR normal, the event rate was low. Conversely, when the MBF was normal but CFR abnormal, the risk was intermediate. Explanations for this have been suggested. For example, it has been suggested that CFR/MPR may be measuring the vasodilator capacity, which may be more important than peak MBF. An alternative suggestion is that there are biases and systematic errors in the stress and rest MBF, which are eliminated when measuring MPR. Another potential confounder is that the most common tracer used in the studies is rubidium, in which the extraction fraction is lower than ^15^O-water PET, and this might affect precision at hyperemic flow measurements.

The mechanism for impaired myocardial perfusion contributing to worse outcomes is likely to be a combination of epicardial coronary disease and microvascular dysfunction. Standard perfusion images rely on the assumption that there is a “normal vessel” that supplies the reference myocardium. This may result in the underestimation of impaired perfusion, which may contribute to poor outcome even in patients without perfusion defects. In diffuse epicardial disease, the ability for vasodilation may be impaired, which can cause a continuous pressure fall along an artery, likely contributing to ischemia in the absence of focal disease on angiography.^34^ Also, impaired perfusion in the absence of significant epicardial disease has been associated with increased microvascular resistance caused by microvascular dysfunction.^22,35^ In our cohort, patients with lower MBF and MPR had more cardiac risk factors. This suggests that these conditions are associated with an impairment of myocardial perfusion. Whether MACE is associated with microvascular or macrovascular disease or a combination of both in our cohort is unclear.

### Limitations

With the relatively low event rate and large number of covariates, there is a potential for bias in the estimated coefficients. However, a sensitivity analysis with the Firth penalized model was used to check for bias. The conclusions were the same for both models, making this bias unlikely. This is an observational trial, and as such, the associations reported do not necessarily imply causation. Although many potential confounders were adjusted for, it is possible that an unmeasured or incompletely accommodated confounding factor may have influenced the results. Furthermore, as the study used electronic documentation to acquire outcome data, it is possible that a small number of events were missed. These limitations are consistent with previous perfusion outcome studies. We did not include cause of death in this study because this was not available from the UK Office for National Statistics and may be prone to misclassification bias. Myocardial perfusion is likely to be more strongly associated with cardiovascular causes of death than all-cause mortality as used in this study. Although the perfusion mapping technique is robust, there were errors in 1.1% of cases. Errors can occur because of failures with motion correction, incorrect identification of the left ventricular blood and mistriggering. However, quality control images (such as blood pool identification, arterial input function graphs, and heart rate triggers) are output on the scanner in addition to the perfusion maps. This enables the clinician to have confidence in the quality of data used to produce the map.

## Conclusions

Quantitative CMR perfusion mapping with automatic inline flow measurement using an artificial intelligence approach permits the clinical use of myocardial perfusion at scale. Here, in a 2-center outcome study, the largest such study to date, both stress MBF and MPR were associated with death and MACE independently of other clinical risk markers. This provides the basis to use routinely acquired MBF and MPR to target therapy, which will require validation in prospective randomized controlled trials.

## Sources of Funding

Dr Knott is supported by a British Heart Foundation Clinical Research Training Fellowship (FS 17/34/32901). This work forms part of the research areas contributing to the translational research portfolio of the Biomedical Research Centre at Barts, which is supported and funded by the National Institute for Health Research. Dr Davies was funded through the Capital Enterprise - Artifical Intelligence program by a grant from the European Regional Development Fund and Barts Charity.

## Disclosures

Dr Petersen provides consultancy and has stock options for Circle Cardiovascular Imaging Inc, Calgary, Alberta, Canada. The other authors report no conflicts.

## Supplementary Material


