# Supplementary material for: The Prognostic Significance of Quantitative Myocardial Perfusion: An Artificial Intelligence–Based Approach Using Perfusion Mapping
Source: Circulation. 2020 Feb 14;141(16):1282–91. doi: 10.1161/CIRCULATIONAHA.119.044666 (PMC7176346; doi:10.1161/CIRCULATIONAHA.119.044666)
Supplement: Supplementary file 1 [file cir-141-1282-s001.pdf]

Supplemental Material

	1.5T (n=679)	3.0T (n=370)	Significance (p value)
Stress MBF (ml/g/min)	2.07	2.06	0.807
MPR	2.49	2.47	0.801
Death, n (%)	25 (3.7)	17 (4.6)	0.511
MACE, n (%)	116 (17.1)	58 (15.7)	0.603

Supplemental Table 1. Comparison of perfusion data by field strength. Abbreviations: Myocardial blood flow (MBF), myocardial perfusion reserve (MPR), major adverse cardiovascular events (MACE), Tesla (T).

	MACE	No MACE	Significance (p value)
Stress MBF 1.5T (ml/g/min)	1.61	2.16	**<0.0001**
MPR 1.5T	1.94	2.60	**<0.0001**
Stress MBF 3.0T (ml/g/min)	1.64	2.13	**<0.0001**
MPR 3.0T	2.24	2.52	**0.020**
BHC MBF (ml/g/min)	1.58	2.11	**<0.0001**
BHC MPR	2.06	2.54	**<0.0001**
RFH MBF (ml/g/min)	1.76	2.41	**<0.0001**
RFH MPR	1.97	2.72	**<0.0001**

Supplemental Table 2. Comparison of MACE events by field strength and scanning site. Abbreviations: Myocardial blood flow (MBF), myocardial perfusion reserve (MPR), major adverse cardiovascular events (MACE), Tesla (T), Barts Heart Centre (BHC), Royal Free Hospital (RFH).
